# Chemical Composition of the Moss *Polytrichum Commune*: A Focus on Structural Biopolymers

**DOI:** 10.3390/plants15142154

**Published:** 2026-07-13

**Authors:** Nikolay A. Budaev, Artyom V. Belesov, Ilya I. Pikovskoi, Anna V. Faleva, Alexey V. Malkov, Sergey A. Pokryshkin, Evgeniy A. Toptunov, Nikolay V. Ul’yanovskii, Dmitry S. Kosyakov

**Affiliations:** 1 Core Facility Center ‘Arktika’, Northern (Arctic) Federal University, Arkhangelsk 163002, Russia; 2 Innovative Facilities Engineering and Innovation Center ‘Advanced Northern Bioresources Processing Technologies’, Northern (Arctic) Federal University, Arkhangelsk 163002, Russia

**Keywords:** *Polytrichum commune*, moss, biopolymers, cellulose, hemicellulose, polyphenols

## Abstract

The biomass of *Polytrichum commune* Hedw. (Polytrichaceae) moss demonstrates high pharmaceutical and biorefining potential, but its main structural biopolymers (cellulose, hemicellulose, and polyphenolic compounds) remain incompletely characterized. In this work, the method of nitric acid–ethanol extraction and dioxane extraction (Pepper’s method) was used to isolate cellulose and lignin. XRD, SEC, FTIR, and Py-GC/MS were used for the biopolymer’s characterization. The composition of hemicellulose was determined without prior isolation using HPLC analysis of the total biomass hydrolysate. The yields were: cellulose 23.0 ± 0.7%, hemicellulose (calculated as sum of mannose, galactose and xylose) 24.1 ± 0.5%, and dioxane-extractable polyphenolic fraction 8.0 ± 0.4% of dry biomass. The cellulose exhibits a low degree of polymerization (360) and moderate crystallinity (40.4% as determined by XRD). The hemicellulose is dominated by mannose and galactose (60% and 30% of non-cellulosic sugars), indicative of a galactomannan. The dioxane-extractable polyphenolic fraction has a low molecular weight (M_n_~1100 Da) and an atypical H:S:G monomer ratio of 58:35:7, confirming its condensed non-lignin structure. These properties of moss biopolymers offer opportunities for comprehensive biomass processing and for further pharmacological evaluation of the polyphenolic fraction as a potential antioxidant source.

## 1. Introduction

The global transition from a fossil-based linear economy to a circular bioeconomy depends on sustainable technologies for valorizing underutilized renewable feedstocks [1,2]. Lignocellulosic biomass from conventional agriculture and forestry has been extensively investigated for bio-based products [3], but non-wood biomass is also considered a source of valuable compounds and materials [4]. Bryophytes (mosses) are one of the oldest lineages of land plants and dominate the ground cover of boreal and subarctic ecosystems [5,6]. Moss biomass has potential value for biorefining and, due to its rich secondary metabolism, for pharmaceutical applications.

Mosses are the second largest group in the plant kingdom after flowering plants, with more than 20,000 species. They grow on all continents and comprise about 5% of all plant species on Earth [7]. The main groups of bioactive secondary metabolites in mosses are terpenes, steroids, cyanoglycosides, and various aromatic (phenolic) compounds [8]. Phenolic compounds are the most extensive group and include flavonoids and their glycosides, bibenzyl and bis(bibenzyl) derivatives, alkyl and aryl benzoates, coumarins, and monomeric aromatic acids (benzoic, protocatechuic, vanillic, ferulic, and caffeic) [9,10,11]. The applicability of moss biomass in biorefinery contexts has received increasing attention [12,13].

Among mosses, *Polytrichum commune* (common haircap moss) is widespread in temperate and boreal latitudes of the Northern Hemisphere. Its stems reach 50 cm in favourable conditions, making *P. commune* the largest moss in the world, and its biomass is readily available for harvesting. In folk medicine, *P. commune* has been used as an antipyretic and antidote to stop bleeding and to treat cuts, pneumonia, and pulmonary tuberculosis [14,15]. In Chinese traditional herbal medicine, the dried whole plant is used to treat memory decline and other related diseases [16]. The moss biomass contains terpenoids, flavonoids, and hybrid molecules such as the flavanone–styryl communins and benzonaphthoxanthenones (e.g., ohioensin) [16,17,18]. Recent phytochemical work revealed novel oligomeric dihydrochalcones and a unique dihydrocinnamoyl bibenzyl, polycommunin A [19,20]. Comprehensive phytochemical studies on other medicinal plant genera, such as *Trigonostemon chinensis* Merr. (Euphorbiaceae) [21] and *Ilex hainanensis* Merr. (Aquifoliaceae) [22], have demonstrated the importance of systematic characterization of bioactive constituents for linking traditional uses to pharmacological activities. This underscores the need for detailed chemical profiling of *P. commune* biopolymers and secondary metabolites.

The above-mentioned features support the traditional use of *P. commune* and suggest potential for pharmaceutical, nutraceutical and cosmeceutical applications [23,24]. The presence of lignin or lignin-like substances in mosses has long been debated. Early studies reported lignin in some giant mosses [25], but later work refuted this [26]. Karmanov et al. described a lignin from *P. commune* similar to softwood lignin [27]. However, a recent study by Rencoret et al. showed that the dioxane-extractable fraction of *P. commune* is not true lignin, with only weak flavonoid signals detected [28]. In our previous work, we characterized the extractable polyphenolic fractions of *P. commune* and identified new antioxidant compounds [19,20]. However, a systematic characterization of the major structural biopolymers (cellulose, hemicellulose, and the dioxane-extractable fraction) from the same batch of biomass has not yet been performed.

Beyond traditional medicine, *P. commune* biomass has been explored for environmental monitoring (heavy metal accumulation), as a soil amendment in peatland restoration [29,30], and as a source of bioactive secondary metabolites for cosmetic and nutraceutical formulations [14,15,16,17]. However, the structural biopolymers that constitute the bulk of the biomass have received much less attention, limiting the development of integrated biorefinery schemes. Although individual reports exist on lignin-like fractions [27] or extractable phenolics [19,20], a systematic characterization of all three major structural biopolymers (cellulose, hemicellulose, and the dioxane-soluble polyphenolic material) from the same biomass batch has not been performed. In particular, the degree of polymerization and crystallinity of the moss cellulose, the monosaccharide profile of its hemicellulose, and the molecular-weight distribution and monomeric composition of its polyphenolic fraction remain unknown. This work fills that gap.

In the present study, we characterize cellulose, hemicellulose and the polyphenolic fraction isolated from a single batch of *P. commune* biomass. We isolated cellulose by a nitric acid–ethanol procedure and the polyphenolic fraction by dioxane extraction according to Pepper’s method [31]. We determined hemicellulose composition by HPLC analysis of the total biomass hydrolysate without prior isolation [32]. Using XRD, SEC, FTIR, and Py-GC/MS, we determined the degree of polymerization and crystallinity of the moss cellulose, the monomeric composition of its hemicellulose, and the molecular weight and monomeric profile of the dioxane-extractable polyphenolic fraction. We also assessed the potential suitability of each biopolymer for pharmaceutical and biorefinery applications. This work fills a critical gap in the phytochemistry of bryophytes and guides the development of selective fractionation strategies for this medicinally promising biomass.

## 2. Results and Discussion

### 2.1. Isolation and Characterization of Cellulose

Cellulose was isolated from *P. commune* biomass with a yield of 23.0 ± 0.7% based on dry weight. The α-cellulose content of the preparation was 83.7%, confirming a low content of alkali-soluble polysaccharides and, therefore, a high purity of the isolated cellulose. The average degree of polymerization of this cellulose was 360, which is a value substantially lower than the DP characteristic of softwood or cotton cellulose (800–1500) [33]. It should be noted that the nitric acid–ethanol treatment may cause partial depolymerization of cellulose, potentially leading to a slightly lower measured DP compared to native cellulose.

**Figure 1:**
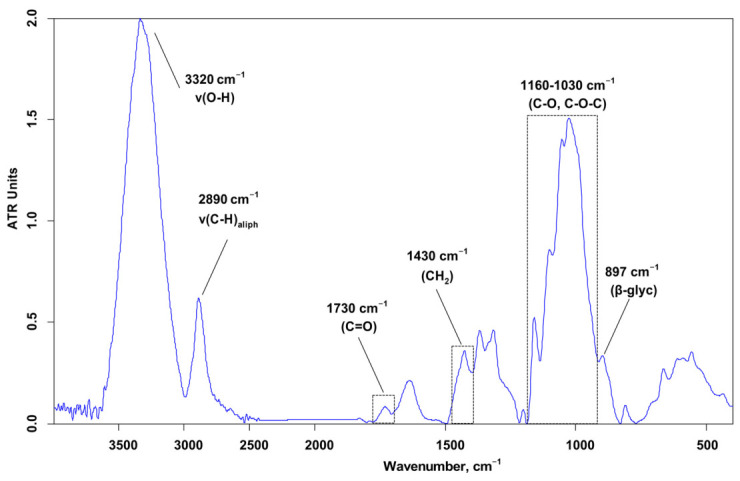
FTIR spectrum of cellulose isolated from *P. commune*. Major bands: 3320 (O-H stretch), 2890 (C-H), 1430 (CH_2_ bending, crystalline), 1160–1030 (C-O-C and C-O of pyranose ring), 897 cm^−1^ (β-glycosidic linkage, amorphous).

The FTIR spectrum of isolated cellulose (Figure 1) exhibits bands characteristic of cellulose I. A strong, broad O-H stretching band at 3339 cm^−1^ was accompanied by aliphatic C-H stretching at 2891 cm^−1^. The region 1430–1315 cm^−1^ contains CH_2_ bending (1427 cm^−1^), C-H deformation (1369 cm^−1^), and O-H in-plane bending (1334 cm^−1^) bands. The complex absorption at 1200–950 cm^−1^ is dominated by intense bands at 1159, 1101, 1053, and 1028 cm^−1^. They arise from C-O-C and C-O stretching of the pyranose ring and glycosidic linkages. The band at 897 cm^−1^ is diagnostic of β-glycosidic bonds and is characteristic of amorphous cellulose. Its intensity is comparable to that of the 1427 cm^−1^ band (crystalline-sensitive), indicating a moderate degree of crystallinity. A weak absorption at 1733 cm^−1^ indicates minor hemicellulose contamination. The overall spectral profile is consistent with that reported for cellulose from the bryophytes [34] and for brown algal cellulose [35], but it differs from highly crystalline cotton cellulose by the relatively intense 897 cm^−1^ band, reflecting a higher proportion of amorphous regions.

**Figure 2:**
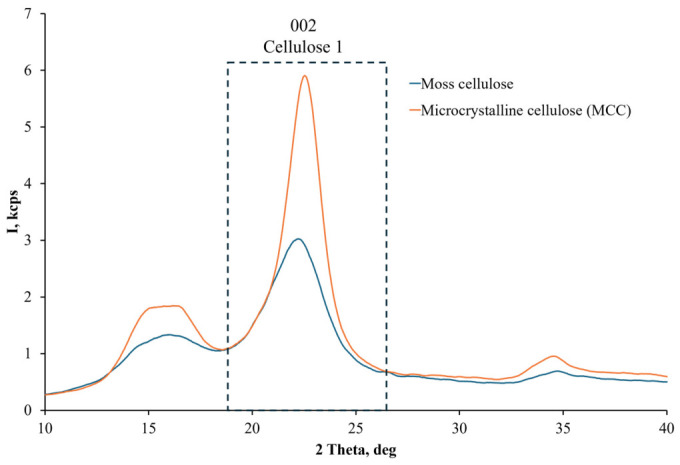
XRD patterns (Cu Kα, 50 kV, 30 mA, 2θ range 10–70°) of moss cellulose and microcrystalline cellulose reference.

XRD analysis confirmed the FTIR findings. The diffraction pattern of the moss cellulose (Figure 2) exhibited reflections at 2θ ≈ 15° and 22.6°, which are identical to those of the type I polymorph of cellulose and matched the pattern of a commercial microcrystalline cellulose (MCC) reference [36,37]. Crystallinity values determined from the XRD data are presented in Table 1. Using the internal Shimadzu v. 5.0 software (based on the Hermans–Weidinger method), the crystallinity was 40.4 ± 0.1% for moss cellulose and 47.8 ± 0.5% for MCC, respectively. Using the same diffraction data with ^13^C NMR data for calibration, the values were 35.5 ± 0.2% and 50.7 ± 1.0%. Thus, the moss cellulose is a moderately crystalline cellulose I.

The discrepancy between the two crystallinity values arises from the different physical principles. The Shimadzu software empirically separates crystalline and amorphous contributions by peak integration, whereas the NMR-calibrated method accounts for molecular mobility of cellulose chains. Both approaches consistently indicate moderate crystallinity, which is lower than that of typical wood cellulose.

The moderate crystallinity (35–40%) and low degree of polymerization (360) demonstrate that this cellulose is more susceptible to enzymatic hydrolysis than typical high-DP, highly crystalline wood cellulose. This is consistent with comparative studies demonstrating faster hydrolysis rates for low-DP celluloses [33,38]. Also, it is possible to use cellulose preparations as biologically active additives, as well as raw materials for the production of medical hydrogels [39,40]. Thus, besides biomedical applications, the moss cellulose is a candidate for processes in which rapid saccharification is a priority, such as the production of fermentable sugars or bioethanol.

### 2.2. Monosaccharide Composition of Hemicellulose

Based on the monosaccharide composition of the total biomass hydrolysate (Table 2), the hemicellulose content was calculated as the sum of mannose, galactose, and xylose. This gave 240.7 mg⋅g^−1^ or 24.1 ± 0.5% of the dry biomass.

The hydrolysate contained glucose as the most abundant monomer, which originated predominantly from cellulose. Among the non-cellulosic sugars, mannose (~60%) and galactose (~30%) were the main constituents, while xylose accounted for ~10%. Arabinose was below the limit of quantitation, while fructose and apiose were only minor components.

It should be noted that the hemicellulose composition was inferred from the total biomass hydrolysate without prior isolation of individual fractions, so a minor contribution from amorphous cellulose or other β-glucans cannot be completely excluded. Nevertheless, the dominance of mannose and galactose over xylose is a robust diagnostic feature of a galactomannan-type hemicellulose.

This monosaccharide profile is characteristic of a galactomannan (or xylogalactomannan) hemicellulose. This composition resembles the polysaccharide profiles of green algae [41,42]. Also, this composition is consistent with the hemicellulose profiles reported for other bryophytes. In the moss *Physcomitrium patens* (Hedw.) Bruch & Schimp. (Funariaceae), mannans are the major hemicellulose in all tissues [43]. In *Hypnodendron menziesii* (Hook.) Par. (Hypnodendraceae), heteromannans (probably galactoglucomannans) predominate in gametophytic stems [44]. The *Marchantia polymorpha* L. (Marchantiaceae) also contains mannans, including galactomannans, along with xyloglucans and xylans [45]. Thus, galactomannan-type hemicelluloses appear to be a conserved feature of bryophyte cell walls, distinguishing them from the xylose-rich hemicelluloses of many vascular plants. Arabinose and apiose were barely detectable. Thus, pectic polysaccharides are not significant. The predominance of galactomannans enables the use of moss hemicelluloses in biomedicine for intraarticular injection and, following modification, for drug delivery applications [46,47,48,49].

### 2.3. Characterization of the Dioxane-Extractable Polyphenolic Fraction

**Figure 3:**
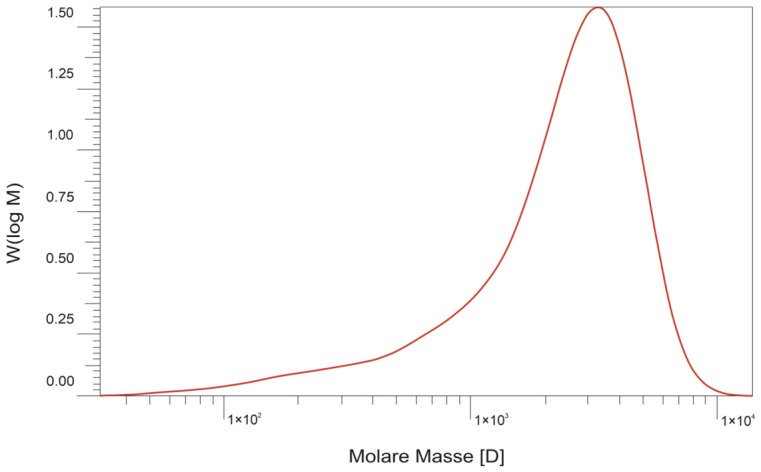
SEC chromatogram (UV detection at 275 nm) of the dioxane-extractable polyphenolic fraction. Separation on an MCX column at 40 °C with 0.1 M NaOH as a mobile phase. Calibration with sodium poly(styrene sulfonate) standards with a mass range of 1.3–200 kDa.

The dioxane-extractable polyphenolic fraction constituted 8.0 ± 0.4% of the dry biomass. This accounted for ~30% of the total polyphenol content (Klason lignin 25.0 ± 0.8%). Its molecular weight, determined by SEC, was relatively low, with M_n_ ≈ 1.1 kDa, M_w_ ≈ 2.7 kDa, and a polydispersity index of 2.4 (Figure 3). Monomodal distribution is characteristic of oligomeric polyphenol preparations rather than high-molecular-weight lignins [50].

**Figure 4:**
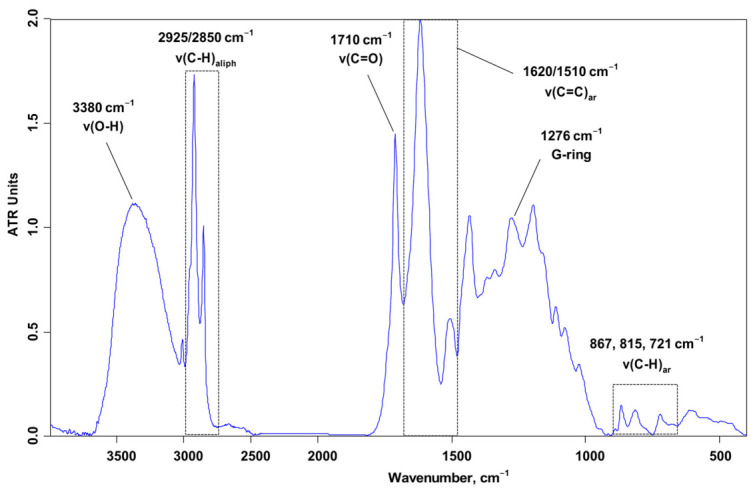
FTIR spectrum of the dioxane-extractable polyphenolic fraction from *P. commune*. Major bands: 3380 cm^−1^ (O-H stretch), 2925 and 2855 cm^−1^ (aliphatic C-H), 1710 cm^−1^ (C=O), 1620 and 1510 cm^−1^ (aromatic skeletal vibrations), 1276 cm^−1^ (guaiacyl ring breathing), 1200–1000 cm^−1^ (C-O of phenolic, ether and alcohol groups), and 867, 815, 721 cm^−1^ (out-of-plane C-H deformation).

The FTIR spectrum (Figure 4) displayed features characteristic of a hydroxylated aromatic polymer but differed in several respects from typical softwood and hardwood lignin. A broad O-H stretching band centered at ~3360 cm^−1^, aliphatic C-H stretches at 2924 and 2854 cm^−1^, and a carbonyl band at 1711 cm^−1^ indicated the presence of hydroxyl, alkyl, and carbonyl functional groups. Aromatic skeletal vibrations at 1617, 1506 cm^−1^ and out-of-plane C-H deformation bands at 867, 815, and 721 cm^−1^ confirmed the occurrence of substituted aromatic rings.

Absorptions in the range 1200–1000 cm^−1^ arise from C-O stretching of phenolic, ether, and alcohol groups, with maxima at 1195, 1112, 1079, and 1024 cm^−1^. The 1276 cm^−1^ band, assigned to guaiacyl ring breathing, is moderate but less intense relative to the aromatic skeletal bands (1616, 1506 cm^−1^) than in typical softwood lignins [28]. The overall spectral envelope is dominated by a prominent carbonyl band at 1710 cm^−1^ and a relatively weak guaiacyl signal, which distinguishes this fraction from authentic softwood or hardwood lignins and is consistent with the condensed non-lignin polyphenolic structures reported for dioxane extracts of mosses [28].

**Figure 5:**
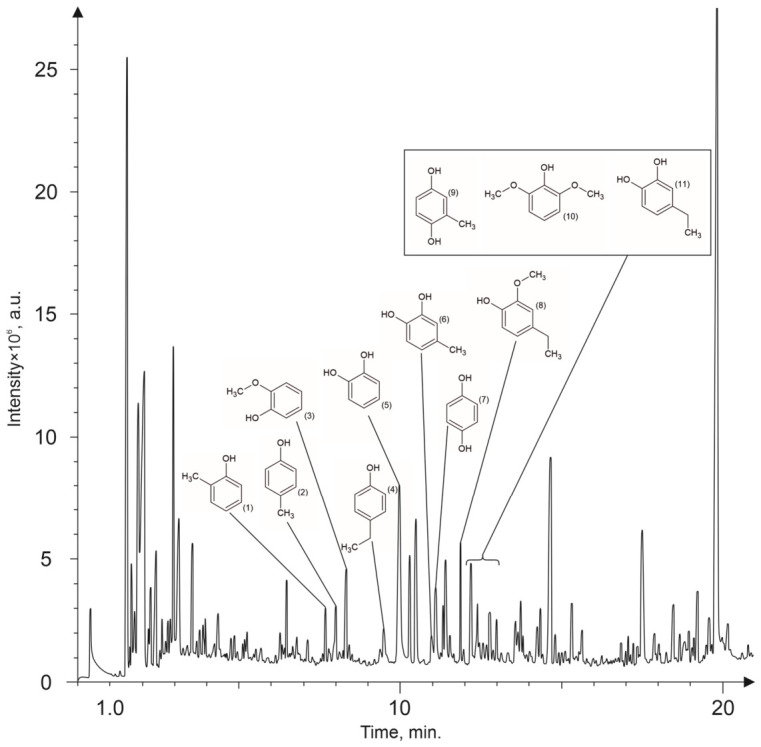
Py-GC/MS pyrogram of the dioxane-extractable polyphenolic fraction. Peaks 1–11 are key phenolic markers: 1—o-cresol, 2—p-cresol, 3—guaiacol, 4—4-ethylphenol, 5—catechol, 6—4-methylcatechol, 7—hydroquinone, 8—ethylguaiacol, 9—methylhydroquinone, 10—syringol, 11—4-ethylcatechol. Identification confidence > 85% (NIST/Wiley 2011). Full list in Table S1.

Py-GC/MS revealed the monomer composition (Figure 5) of the polyphenolic fraction. The obtained pyrogram contained peaks of a complex mixture of products (Table S1). The eleven most diagnostically significant phenolic compounds, labeled on the chromatogram and accompanied by their structural formulae, were o-cresol (1), p-cresol (2), guaiacol (3), 4-ethylphenol (4), catechol (5), 4-methylcatechol (6), hydroquinone (7), ethylguaiacol (8), methylhydroquinone (9), syringol (10), and 4-ethylcatechol (11).

The relative monomeric composition, the H:S:G ratio calculated from the sum peak areas of the assigned markers, was 58:35:7. This approach provides a semi-quantitative estimate of the monomeric composition. This distribution is not typical of softwood, hardwood, or grass lignin [26]. This monomer profile is not consistent with the canonical monolignol pathway. Instead, it suggests a polyphenol rich in ortho- and para-hydroxylated rings. Taken together, the SEC, FTIR and Py-GC/MS results provide evidence that the dioxane-extractable polyphenolic fraction of *P. commune* is a condensed non-lignin polyphenol.

The high content of phenolic hydroxyl groups and the abundance of catechol-type substructures suggest that this condensed polyphenol may be of interest for further antioxidant activity screening and pharmacological evaluation, although direct bioactivity assays are required to confirm these potential applications [16,19,20]. However, a detailed structural characterization of this fraction, including the identification of inter-unit linkages and the full molecular architecture, requires a separate investigation employing high-resolution mass spectrometry and two-dimensional nuclear magnetic resonance spectroscopy, which will be the subject of a forthcoming study.

In summary, the combined spectroscopic and chromatographic data classify the dioxane extract as a non-lignin condensed polyphenolic oligomer. This is consistent with the findings of Rencoret et al. [28], who reported that dioxane extracts from mosses lack the typical monolignol-derived inter-unit linkages. The dominance of catechol-type substructures (peaks 5, 6, 11 in Figure 5) and the high proportion of p-hydroxyphenyl units (H-type, 58%) suggest a biosynthetic pathway distinct from that of vascular plant lignins. This structural peculiarity may explain the relatively low molecular weight (apparent M_n_ ≈ 1.1 kDa) and the high carbonyl content (1711 cm^−1^), which are atypical for wood lignins.

## 3. Materials and Methods

### 3.1. Materials and Sample Preparation

*Polytrichum commune* moss was collected in September 2024 from a swampy boreal forest in the Primorsky district of the Arkhangelsk region, Russia. GPS coordinates of the three sampling points were: 64.412247, 40.540506; 64.411928, 40.539527; and 64.412121, 40.540283. Soil analysis was not performed in this study. The moss was identified as *Polytrichum commune* by morphological examination using a voucher specimen (No. NARFU-2024-09-001) of the Herbarium of Northern (Arctic) Federal University.

Biomass was collected from three separate colonies (*n* = 3) located 20–40 m apart. All analyses were performed on each biological replicate in duplicate (technical replicates). Results are expressed as mean ± standard deviation (SD) of the biological replicates.

The plant material was air-dried at room temperature and stored in airtight containers in the dark for 2–3 weeks before further processing. The dry biomass was ground using an ultracentrifugal mill ZM 200 (Retsch, Haan, Germany) equipped with a 1 mm sieve. The moisture content of the dry raw material (7.5%) was determined with a moisture analyzer HG63 (Mettler Toledo, Columbus, OH, USA). A photograph of the collection site and the moss before harvesting is provided in Figure S1.

### 3.2. Isolation of Cellulose

Cellulose was isolated from 1 g of ground moss by a nitric acid–ethanol method [51]. The sample was refluxed with 100 mL of a nitric acid–ethanol mixture (1:4) in a 250 mL conical flask on a boiling water bath for 1 h. After filtration, the residue was washed with 25 mL of the same acid–ethanol mixture. The boiling–filtration cycle was repeated four times. The final residue was thoroughly washed with hot distilled water until neutral pH and dried under vacuum to a constant weight. All yield determinations were performed in triplicate (*n* = 3 biological replicates, each measured in duplicate).

### 3.3. Isolation of the Polyphenolic Fraction

The polyphenolic fraction was isolated as dioxane lignin according to Pepper’s method [31]. Air-dried moss was pre-extracted with acetone in a Soxhlet apparatus to remove extractives. The pre-extracted material was then subjected to acidolytic dioxane extraction (0.2 M HCl in dioxane–water mixture, 9:1) under a nitrogen atmosphere. The preparation was purified by reprecipitation from dioxane solution into diethyl ether. The yield of the dioxane-extractable fraction was 8.0 ± 0.4% of the pre-extracted moss.

To determine the total content of polyaromatic compounds, the biomass was treated with concentrated and diluted sulfuric acid (Klason lignin), followed by gravimetric determination of the proportion of the acid-resistant part. The yield of Klason lignin preparations was 25.0 ± 0.6% of the pre-extracted moss.

### 3.4. Determination of α-Cellulose Content

The content of α-cellulose in the isolated cellulose was determined by a standard alkali extraction procedure. A 3 g portion of cellulose was placed in a 150 mL porcelain beaker, and 45 mL of 17.5% sodium hydroxide solution was gradually added at 20 °C. After 45 min, 45 mL of distilled water was added, and the mixture was stirred. The cellulose residue was collected by vacuum filtration, washed successively with 25 mL of 9.5% NaOH and a large volume of distilled water until neutral, and then dried at 105 °C to a constant weight. The α-cellulose content was calculated as the percentage of the initial weight. The α-cellulose content was determined in two parallel experiments plus one control (total *n* = 3).

### 3.5. Determination of the Degree of Polymerization of Cellulose

The average degree of polymerization (DP) of cellulose was determined by the viscometric method in accordance with [52]. A weighed sample of cellulose was dissolved in cadoxene, and the intrinsic viscosity of the resulting solution was measured by recording the efflux time in a VPZh-3 capillary viscometer (EKROS, Saint-Petersburg, Russia) after thermostating. The DP value was calculated from the intrinsic viscosity.

### 3.6. X-Ray Diffraction Analysis

X-ray diffraction (XRD) patterns of the cellulose sample and a reference microcrystalline cellulose (MCC; Sigma–Aldrich, St. Louis, MO, USA) were recorded on a Shimadzu XRD-7000 S diffractometer (Shimadzu, Kyoto, Japan) equipped with a Cu Kα source (λ = 1.5406 Å) operating at 50 kV and 30 mA. The samples were pressed into tablets (diameter 25 mm, thickness 1.0 mm) under a load of 10 t. Diffractograms were acquired in reflection mode on a non-reflecting silicon holder with sample rotation (30 rpm) over the 2θ range 10–70°, with a step size of 0.020° and a scanning speed of 0.5°·min^−1^. Two parallel measurements were performed.

### 3.7. Monosaccharide Composition Analysis

The monosaccharide composition of the total biomass was determined by HPLC analysis of the acid hydrolysate without prior isolation of the polysaccharide fractions [32]. A 10 mg sample was treated with 100 μL of 72% H_2_SO_4_ in a 4 mL conical vial at 30 °C for 60 min using a Reacti-Therm TS-18821 heating module (Thermo Scientific, Waltham, MA, USA). Subsequently, 2.5 mL of deionized water was added, and the hydrolysis was continued at 100 °C for 3 h with constant stirring. After cooling, the hydrolysate was neutralized with BaCO_3_, and the completion of neutralization was checked with litmus paper. The resulting solution was filtered through a 0.22 μm nylon membrane filter.

Chromatographic analysis was carried out on a Nexera HPLC system (Shimadzu, Kyoto, Japan) consisting of a vacuum degasser, three pumps, an autosampler, a column thermostat, and a refractive index detector. Separation was performed on a Rezex RPM-Monosaccharide Pb^2+^ column (300 × 7.8 mm; Phenomenex, Torrance, CA, USA) at 75 °C, using deionized water (resistivity 18.2 MΩ·cm) as the mobile phase at a flow rate of 0.6 mL·min^−1^. The injection volume was 10 μL, and the total run time was 40 min. Data acquisition and processing were carried out with LabSolution software ver. 5.99.

Hemicellulose content was calculated as the sum of mannose, galactose and xylose using factors of 0.90 for hexoses and 0.88 for pentoses.

### 3.8. Size-Exclusion Chromatography of the Polyphenolic Fraction

The number-average (M_n_) and weight-average (M_w_) molecular weights of the dioxane-extractable polyphenolic fraction were determined by size-exclusion chromatography (SEC) on an LC-20 Prominence HPLC system (Shimadzu, Kyoto, Japan) equipped with an SPD-20A UV-VIS detector set at 275 nm. An MCX column (300 × 8 mm, pore size 1000 Å; PSS, Esslingen am Neckar, Germany) was used at 40 °C. A 0.1 M sodium hydroxide solution served as both the mobile phase and the sample solvent. The system was calibrated with monodisperse sodium poly(styrene sulfonate) standards (PSS, Esslingen am Neckar, Germany). Because of the different hydrodynamic behaviour of synthetic standards and polyphenolic oligomers, the reported molecular weights are apparent values.

### 3.9. Fourier-Transform Infrared Spectroscopy

Fourier-transform infrared spectra were recorded on a Vertex 70 spectrometer (Bruker, Ettlingen, Germany) equipped with a GladiATR attenuated total reflectance (ATR) accessory (Pike Technologies, Madison, WI, USA). Spectra were collected in absorption mode over the range 4000–400 cm^−1^ at a resolution of 4 cm^−1^ by averaging 128 scans. Data acquisition and processing were performed using OPUS software (Bruker, Ettlingen, Germany) ver. 8.2.28.

### 3.10. Pyrolysis–Gas Chromatography–Mass Spectrometry

Pyrolysis–gas chromatography–mass spectrometry (Py-GC/MS) analysis of the polyphenolic fraction was conducted on a QP-2010Plus GC–MS system (Shimadzu, Kyoto, Japan) coupled to an EGA/PY-3030D microfurnace pyrolizer (Frontier Lab, Fukushima, Japan) equipped with a liquid-nitrogen-cooled cryo-trap. Samples (100–150 μg) were placed in microtubes and thermally decomposed in an inert (helium) atmosphere by heating from 50 to 420 °C at a rate of 150 °C·min^−1^. The pyrolysis temperature of 420 °C was chosen based on preliminary temperature-ramp experiments (300–500 °C) showing maximal release of phenolic fragments with minimal secondary transformation for moss polyphenols. The volatile products were separated on an HP-5ms capillary column (30 m × 0.25 mm i.d., 0.25 μm film thickness; Agilent, Santa Clara, CA, USA) using helium (grade 6.0) as the carrier gas at a constant flow of 1 mL·min^−1^. The column oven was programmed as follows: 40 °C for 2 min, ramp at 10 °C·min^−1^ to 320 °C, and hold for 15 min. Mass spectrometric detection was performed using electron ionization (70 eV) in full-scan mode over the mass range *m*/*z* 15–500 at a scan speed of 3333 Da·s^−1^. Compound identification was achieved by comparison of the acquired mass spectra with the NIST-14/Wiley 2011 spectral libraries.

### 3.11. Statistical Analysis

All results are expressed as mean ± SD from three independent biological replicates (*n* = 3), each measured in duplicate. No inferential statistical tests were applied because the study is descriptive. Confidence intervals are not reported as they would imply population-level inference beyond the scope of this work.

## 4. Conclusions

The main contribution of this work is to provide the first systematic characterization of all three biopolymer fractions (cellulose, hemicellulose and dioxane-extractable polyphenols) from the same *Polytrichum commune* biomass, filling a critical gap in the phytochemistry of bryophytes.

We demonstrated that the moss cellulose has a low degree of polymerization (360) and moderate crystallinity (35–40%). These properties make cellulose suitable for rapid saccharification (e.g., fermentable sugars or bioethanol) and for use as bioactive additives or medical hydrogels.

We have shown that the hemicellulose is a galactomannan type, dominated by mannose (∼60% of non-cellulosic sugars) and galactose (∼30%), with xylose contributing only ∼10%. This composition opens possibilities for direct medical and, after chemical modification, drug delivery applications.

We established that the dioxane-extractable fraction is not a true lignin but a condensed polyphenol with a low molecular weight (M_n_ ≈ 1100 Da) and an atypical H:S:G ratio of 58:35:7. Rich in phenolic hydroxyl and catechol substructures, this fraction is a candidate for further pharmacological evaluation as a potential antioxidant source.

A detailed structural characterization of the polyphenolic fraction, including the identification of inter-unit linkages by 2D HSQC and HMBC NMR, will be the subject of a forthcoming study.

## Figures and Tables

**Table 1:** Crystallinity of moss cellulose and microcrystalline cellulose reference.

Sample	Crystallinity (X_Shimadzu_), %	Crystallinity (X_NMR_), %
Moss cellulose	40.4 ± 0.1	35.5 ± 0.2
MCC	47.8 ± 0.5	50.7 ± 1.0

**Table 2:** Monosaccharide composition of *P. commune* biomass hydrolysate.

No.	Monosaccharide	Content, mg·g^−1^
1	Glucose	217 ± 7
2	Mannose	166 ± 4
3	Galactose	77 ± 3
4	Xylose	25 ± 1
5	Arabinose	<LOQ *
6	Fructose	5.0 ± 0.1
7	Apiose	0.9 ± 0.1

* Limit of Quantitation.

## Data Availability

The data presented in this study are available in the article and Supplementary Materials.

## Supplementary Materials

The following supporting information can be downloaded at: https://www.mdpi.com/article/10.3390/plants15142154/s1, Table S1: Complete list of compounds identified by Py-GC/MS in the dioxane-extractable polyphenolic fraction of *Polytrichum commune*; Figure S1: A photograph of the collection site and the moss before harvesting.

## Abbreviations

The following abbreviations are used in this manuscript:

ATR	Attenuated total reflectance
DP	Degree of polymerization
FTIR	Fourier-transform infrared spectroscopy
H:S:G	Hydroxyphenyl:syringyl:guaiacyl ratio
HPLC	High-performance liquid chromatography
MCC	Microcrystalline cellulose
NMR	Nuclear magnetic resonance
Py-GC/MS	Pyrolysis–gas chromatography/mass spectrometry
SEC	Size-exclusion chromatography
XRD	X-ray diffraction
