# Supplementary material for: Chemical Composition of the Moss Polytrichum Commune: A Focus on Structural Biopolymers
Source: Plants (Basel). 2026 Jul 13;15(14):2154. doi: 10.3390/plants15142154 (PMC13414829; doi:10.3390/plants15142154)
Supplement: Supplementary file 1 [file plants-15-02154-s001.zip › plants-4396022-supplementary.pdf]

Supplementary materials

Chemical Composition of the Moss *Polytrichum Commune*: A Focus on Structural Biopolymers

Nikolay A. Budaev [1], Artyom V. Belesov [1,*], Ilya I. Pikovskoi [1], Anna V. Faleva [1], Alexey V. Malkov [1], Sergey A. Pokryshkin [1], Evgeniy A. Toptunov [2], Nikolay V. Ul'yanovskii [1] and Dmitry S. Kosyakov [1]

[1] Core Facility Center 'Arktika', Northern (Arctic) Federal University, Arkhangelsk 163002, Russia; n.budaev@narfu.ru (N.A.B.); i.pikovskoj@narfu.ru (I.I.P.); a.bezumova@narfu.ru (A.V.F.); a.malkov@narfu.ru (A.V.M.); s.pokryshkin@narfu.ru (S.A.P.); n.ulyanovsky@narfu.ru (N.V.U.); d.kosyakov@narfu.ru (D.S.K.)

[2] Innovative Facilities Engineering and Innovation Center 'Advanced Northern Bioresources Processing Technologies', Northern (Arctic) Federal University, Arkhangelsk 163002, Russia; e.toptunov@narfu.ru

* Correspondence: a.belesov@narfu.ru

Contents:

Table S1. Complete list of compounds identified by Py-GC/MS in the dioxane-extractable polyphenolic fraction of *Polytrichum commune*

Figure S1. A photograph of the collection site and the moss before harvesting.

Table S1. Complete list of compounds identified by Py-GC/MS in the dioxane-extractable polyphenolic fraction of *Polytrichum commune*.

№	Time, min	Area, a.u.	Area, %	Compound
1	1.67	712223	1.3	Naphthalene, 1,2,3,4-tetrahydro-5,8-dimethyl
2	1.77	369938	0.7	Methanol
3	1.88	2393057	94.2	1-propen-2-ol
4	2.05	8291877	14.7	Water
5	2.19	2591394	0.5	5-methyloctene-1
6	2.26	6888826	1.2	Furan, 2-methyl-
7	2.43	12066609	2.1	Acetic acid
8	2.61	2465943	0.4	Benzene
9	2.63	319949	0.1	Butanoic acid, 4-hexenyl ester, (Z)-
10	2.80	1792625	0.3	1-Nonene
11	2.86	1876104	0.3	Heptane
12	2.89	789785	0.1	Propanoic acid
13	2.96	18173492	3.2	1,4-Dioxane
14	3.13	7813871	1.4	Ethanol, 2-ethoxyl-
15	3.55	6602396	1.2	Benzene, methyl-
16	3.78	730222	0.3	1-Octene
17	3.83	184036	0.0	Cyclopentane
18	3.88	987887	0.4	Octane
19	3.96	2499208	0.4	4,8-Dioxaspiro[2.5]oct-1-ene
20	4.35	2291774	0.4	Furfural
21	6.28	1654650	0.3	2-Furancarboxaldehyde, 5-methyl-
22	6.34	13129074	2.3	Ethanol, 2-(2-chloroethoxy)-
23	6.41	1336008	0.2	Hexanoic acid
24	6.48	5911776	1.0	Phenol
25	7.68	3782456	0.7	Phenol, 2-methyl-
26	8.00	3497558	0.6	p-Cresol
27	8.32	6049817	1.1	Phenol, 2-methyl-
28	9.49	2519496	0.5	Phenol, 4-ethyl-
29	9.51	965472	0.2	Octanoic acid
30	9.97	23436727	4.1	Catechol
31	10.29	7707494	1.4	Benzofuran, 2,3-dihydro-
32	10.49	13738210	2.4	5-Hydroxymethylfurfural
33	10.97	1571789	0.3	1,2-Benzenediol, 4-methyl-
34	11.00	1204869	0.2	Nonanoic acid
35	11.10	10404990	1.8	Hydroquinone
36	11.34	3616001	0.6	Phenol, 4-ethyl-2-methoxy-
37	11.40	9519282	1.7	1,2-Benzenediol, 4-methyl-
38	11.87	8868880	1.6	2-Methoxy-4-vinylphenol
39	12.19	9050653	1.6	1,4-Benzenediol, 2-methyl-
40	12.39	3564838	0.6	Phenol, 2,6-dimethoxy-
41	12.78	4339466	0.8	4-Ethylcatechol
42	12.99	2981315	0.5	Tetradecane

43	13.57	2593786	0.5	Benzaldehyde, 2-methoxy-
44	13.74	3795074	0.7	1,2,4-Trimethoxybenzene
45	14.25	2705091	0.5	1-Hexadecanol
46	14.34	3899155	0.7	Tetradecane
47	14.60	1938063	0.3	2-Hydroxy-5-methylisophthalaldehyde
48	14.66	15340121	2.7	Butylhydroxy toluene
49	14.81	1919195	0.3	Benzene, 1,2,3-trimethoxy-5-methyl-
50	15.32	4354210	0.8	3-tert-Butyl-4-hydroxyanisole
51	15.63	1795986	0.3	Tetradecane
52	17.07	1868352	0.3	Heptadecanal
53	17.36	1053135	0.2	5-Chlorovaleric acid, hexadecyl ester
54	17.42	556721	0.1	Ethanone, 1-(4-hydroxy-3,5-dimethoxyphenyl)-
55	17.50	14750608	2.6	1H-Indene-1,5(6H)-dione, 2,3,7,7a-tetrahydro-
56	17.55	3626304	0.6	Tetradecanoic acid
57	18.47	4688914	0.8	2H-1-Benzopyran-2-one, 8-hydroxy-
58	18.67	1907687	0.3	Pentadecanoic acid
59	18.95	1641006	0.3	Neophytadiene
60	19.22	4580665	0.8	Cyclohexane, 2-chloro-4-methyl-1-(1-methylethyl)-
61	19.57	3436989	0.6	7-Tetradecenal, (Z)-
62	19.83	87420532	15.4	n-Hexadecanoic acid
63	21.59	28957789	5.1	9,12-Octadecadienoic acid (Z,Z)-
64	21.65	44197728	7.8	Dichloroacetic acid, tridec-2-ynyl ester
65	21.81	6426845	1.1	Octadecanoic acid
66	22.13	1530495	0.3	Heneicosane
67	23.18	1867657	0.3	Arachidonic acid
68	23.51	4398197	0.8	Cyclohexane, eicosyl-

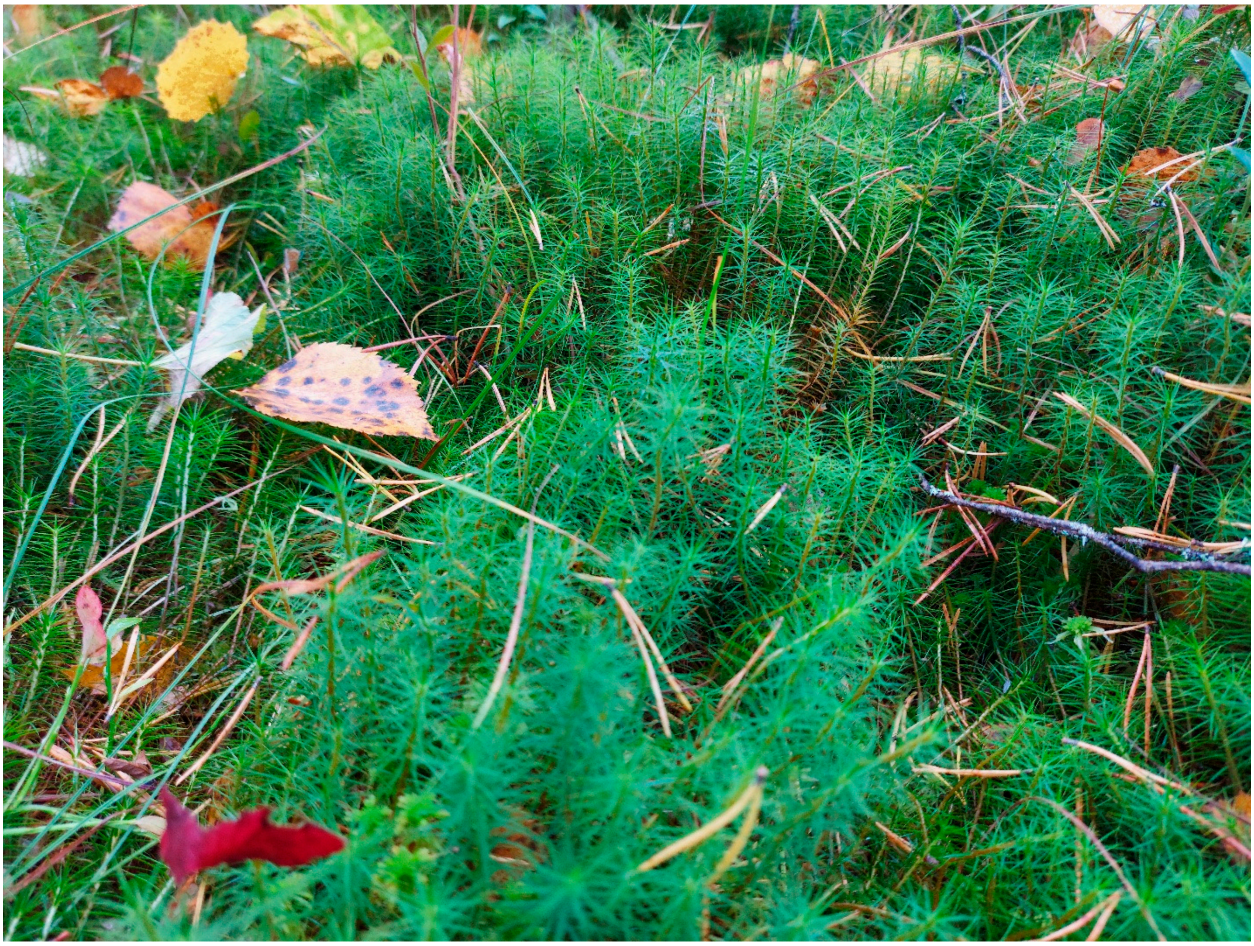
Figure S1. A photograph of the collection site and the moss before harvesting.
